# Chondroitin Sulfate and Proteinoids in Neuron Models

**DOI:** 10.1021/acsabm.4c01678

**Published:** 2025-01-08

**Authors:** Panagiotis Mougkogiannis, Andrew Adamatzky

**Affiliations:** Unconventional Computing Laboratory, University of the West of England, Bristol BS16 1QY, U.K.

**Keywords:** chondroitin sulfate, proteinoids, Izhikevich neuron model, computational neuroscience, neuronal dynamics, extracellular matrix, synaptic plasticity, the Prisoner’s dilemma

## Abstract

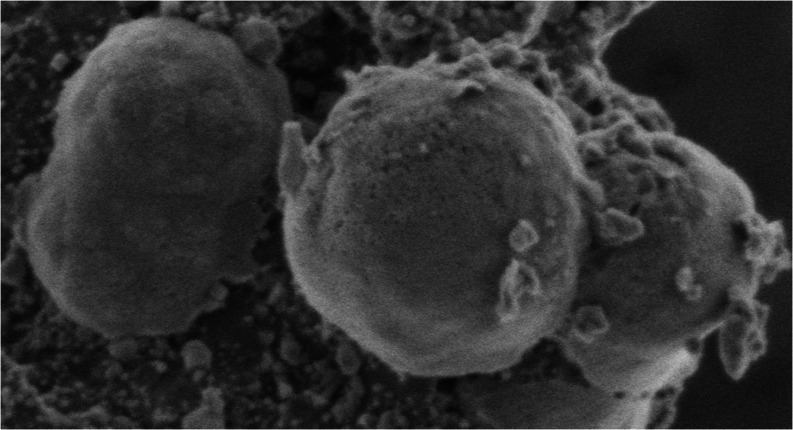

This study examines the relationship between chondroitin sulfate, proteinoids, and computational neuron models, with a specific emphasis on the Izhikevich neuron model. We investigate the effect of chondroitin sulfate-proteinoid complexes on the behavior and dynamics of simulated neurons. Through the use of computational simulations, we provide evidence that these biomolecular components have the power to regulate the responsiveness of neurons, the patterns of their firing, and the ability of their synapses to change within the Izhikevich architecture. The findings suggest that the interactions between chondroitin sulfate and proteinoid cause notable alterations in the dynamics of membrane potential and the timing of spikes. We detect adjustments in the features of neuronal responses, such as shifts in the thresholds for firing, alterations in spike frequency adaptation, and changes to bursting patterns. The findings indicate that chondroitin sulfate and proteinoids may have a role in precisely adjusting neuronal information processing and network behavior. This study offers valuable information about the complex connection between the many components of the extracellular matrix, protein-based structures, and the functioning of neurons. In addition, our analysis of the proteinoid-chondroitine system using game theory uncovers a significant Prisoner’s Dilemma scenario. The system’s inclination toward defection, due to the appeal of cheating and the significant penalty for cooperation, with a mean voltage of −9.19 mV, indicates that defective behaviors may prevail in the long term dynamics of these neuronal interactions.

## Introduction

**Figure 1:**
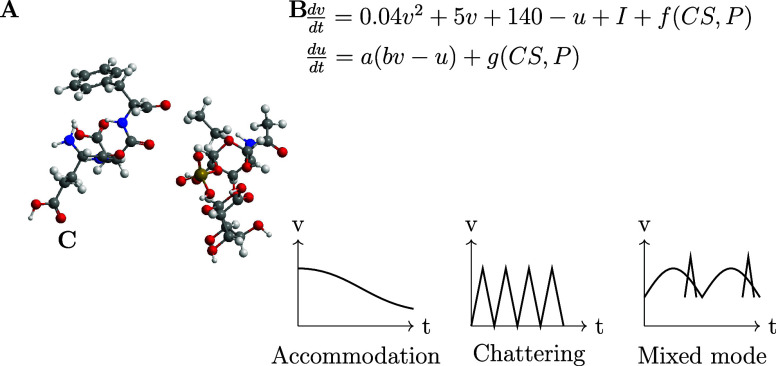
Key concepts in CS-Proteinoid modulation of neuronal dynamics. (A) Structure and interaction of chondroitin sulfate (CS) and proteinoid. (B) Modified Izhikevich model equations incorporating CS-Proteinoid effects. (C) Examples of firing patterns modulated by CS-Proteinoid mixtures: accommodation, chattering, and mixed mode oscillations.

Recent neuroscience research has focused on studying the complex relationship between neuronal dynamics and extracellular matrix components. Two significant modulators of neuronal behavior, chondroitin sulfate (CS) and proteinoids, have emerged from this research. This study aims to understand the complex interactions between CS-proteinoid mixtures and neuronal oscillations, using the Izhikevich neuron model.^1^ Chondroitin sulfate is a glycosaminoglycan that is abundantly found in the brain’s extracellular matrix.^2−5^ It plays important role in neuroplasticity, neuroprotection, and signal transduction.^6^ Proteinoids, which are thermal proteins formed from the thermal polycondensation of mixtures of amino acids, have been used as models for prebiotic protein-like molecules and have demonstrated interesting electrical properties.^7^ The combination of CS and proteinoids creates a unique biomolecular environment that has the potential to significantly influence neuronal behavior. The Izhikevich neuron model, proposed by Eugene Izhikevich in 2003,^8^ offers a computationally efficient and biologically realistic framework for simulating different neural behaviors. The model is defined by a system of two interconnected differential eqs (Figure 1B)
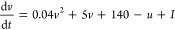
1
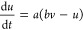
2where *v* represents the membrane potential, *u* is a recovery variable, and *I* is the input current. The parameters *a*, *b*, *c*, and *d* can be adjusted to produce different firing patterns. The objective of our work is to examine the impact of CS-proteinoid mixtures on several neuronal oscillation patterns in this model. The combination of CS and proteinoids creates a unique biomolecular environment that can potentially influence neuronal behavior in profound ways (Figure 1A). These patterns include:

1. Accommodation: The firing rate gradually decreases in response to continuous stimulus.^9^ Our hypothesis suggests that interactions between CS and proteinoids could potentially affect the rate of accommodation by modifying the kinetics of ion channels (Figure 1C, left panel).
2. Chattering: The phenomenon of recurrent episodes of action potentials occurring in a repeated manner.^10^ CS-proteinoid complexes may affect the frequency and length of these bursts (Figure 1C, middle panel).
3. Induced excitability: Neuronal amplification is the process by which a neuron becomes more readily activated as a result of previous stimulation.^11^ We investigate the potential impact of CS-proteinoid mixtures on the threshold for induced excitability.
4. Mixed mode oscillations: The oscillatory patterns include a combination of small and large amplitude oscillations.^12^ CS-proteinoid complexes interacting with neuronal membranes may generate unique mixed mode patterns (Figure 1C, right panel).
5. Phasic spiking: An firing pattern that is distinguished by a solitary spike at the beginning of stimulus.^13^ We examine the potential of CS-proteinoid mixtures to regulate the shift from phasic to tonic firing patterns.

In order to represent these oscillations, we incorporate modifications to the Izhikevich equations to take into account the presence of CS-proteinoid mixtures.
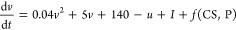
3
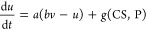
4where *f*(CS, P) and *g*(CS, P) are functions representing the influence of chondroitin sulfate (CS) and proteinoids (P) on membrane potential and recovery dynamics, respectively.

Our study uses the Prisoner’s Dilemma (PD) framework^14−17^ to analyze the interactions between proteinoids and chondroitin in our experimental system. The Prisoner’s Dilemma (PD) is a crucial concept in game theory.^18−20^ It represents scenarios where two individuals face the decision of cooperating or defecting. The payoffs are designed in a way that mutual cooperation leads to the most favorable outcome for both parties. However, individuals are often encouraged to defect in order to maximize their personal gains.^15^ This approach has been extensively used to examine cooperative and competitive behaviors in biological systems, ranging from microbial communities to populations of cancer cells.^21,22^

Our objective is to gain insights into how CS-proteinoid combinations can affect neuronal oscillations in various firing modes by systematically adjusting the parameters of these functions and analyzing the subsequent neuronal behaviors. This research not only enhances our understanding of the complex relationships between extracellular matrix components and neuronal function, but also has implications for neurodegenerative disorders, where changes in CS composition have been detected,^23^ and for the creation of innovative neuroengineering methods.^24^

## Materials and Methods

### Synthesis of Chondroitin Sulfate-Proteinoid Mixture

The chondroitin sulfate-proteinoid mixture was synthesized by combining separate solutions of chondroitin sulfate and proteinoid. Four distinct samples were prepared using varying amounts of chondroitin sulfate (11, 18.6, 40, and 102 mg) dissolved in 5 mL of dimethyl sulfoxide (DMSO) sourced from Sigma-Aldrich (CAS: 67–68–5, EC: 200–664–3, *M*_W_: 78.13 g/mol). Chondroitin sulfate (CAS: 9007–28–7, *M*_W_: 10,000–50,000 g/mol) was accurately weighed using an analytical balance and transferred to clean, dry beakers. The mixtures were then agitated with a magnetic stirrer at room temperature until complete dissolution was achieved. Concurrently, a 5 mL proteinoid solution comprising l-Glutamic Acid (l-Glu), l-Phenylalanine (l-Phe), and l-Aspartic Acid (l-Asp) was prepared in a separate beaker, ensuring thorough dissolution in the aqueous medium. The DMSO-based chondroitin sulfate solutions were then slowly introduced into the aqueous proteinoid solution. The combined solutions were gently mixed using a magnetic stirrer for 5–10 min to ensure complete and uniform blending of the chondroitin sulfate-proteinoid mixtures. Following this process, the synthesized mixtures were ready for subsequent characterization and analysis.

### Electrochemical Characterization Apparatus

**Figure 2:**
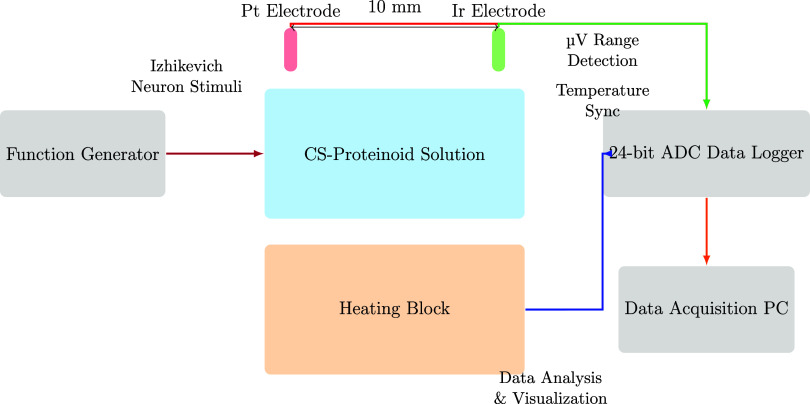
A schematic diagram illustrating the experimental setup used to perform electrochemical characterization of the CS-proteinoid complex. The CS-proteinoid solution with different concentrations of chondroitin sulfate (CS) is contained in the middle container. Two needle electrodes, each plated with platinum (Pt) and iridium (Ir) correspondingly, are placed at a distance of 10 mm from each other in the solution. An ADC data logger with a high level of accuracy, capable of capturing voltage responses in the microvolt (μV) range, is used. Additionally, a heating block is employed to regulate and monitor the temperature. A function generator generates stimuli using Izhikevich neuron models. The data acquisition PC gathers, examines, and presents the system’s responses. This advanced configuration allows for the identification of small changes in voltage and enables a thorough analysis of voltage responses in the CS-proteinoid system.

The voltage responses of the proteinoid-chondroitin sulfate solutions at various concentrations were measured using an electrochemical characterization apparatus, illustrated in Figure 2. This setup comprised a vessel containing the proteinoid-chondroitin sulfate solution, with two needle electrodes (Pt and Ir coated stainless steel wires) inserted at a fixed distance of 10 mm. Voltage responses from these electrodes were captured using a high-precision 24-bit ADC data recorder. To regulate and monitor the solution’s temperature, a heating block was integrated into the apparatus. This feature allowed for the simultaneous recording of both thermal and electrical parameters throughout the characterization process. The ADC data recorder’s exceptional sensitivity enabled the detection of minute voltage fluctuations in the μV range. This capability facilitated the mapping of spatiotemporal voltage responses within the proteinoid-chondroitin sulfate system across different chondroitin sulfate concentrations. In the Supporting Information, there is material characterization, electrical and thermal stability measurements of the CS-proteinoid complex systems.

## Results and Discussion

### Accommodation Spike Analysis of Proteinoid-Chondroitine Sample

**Figure 3:**
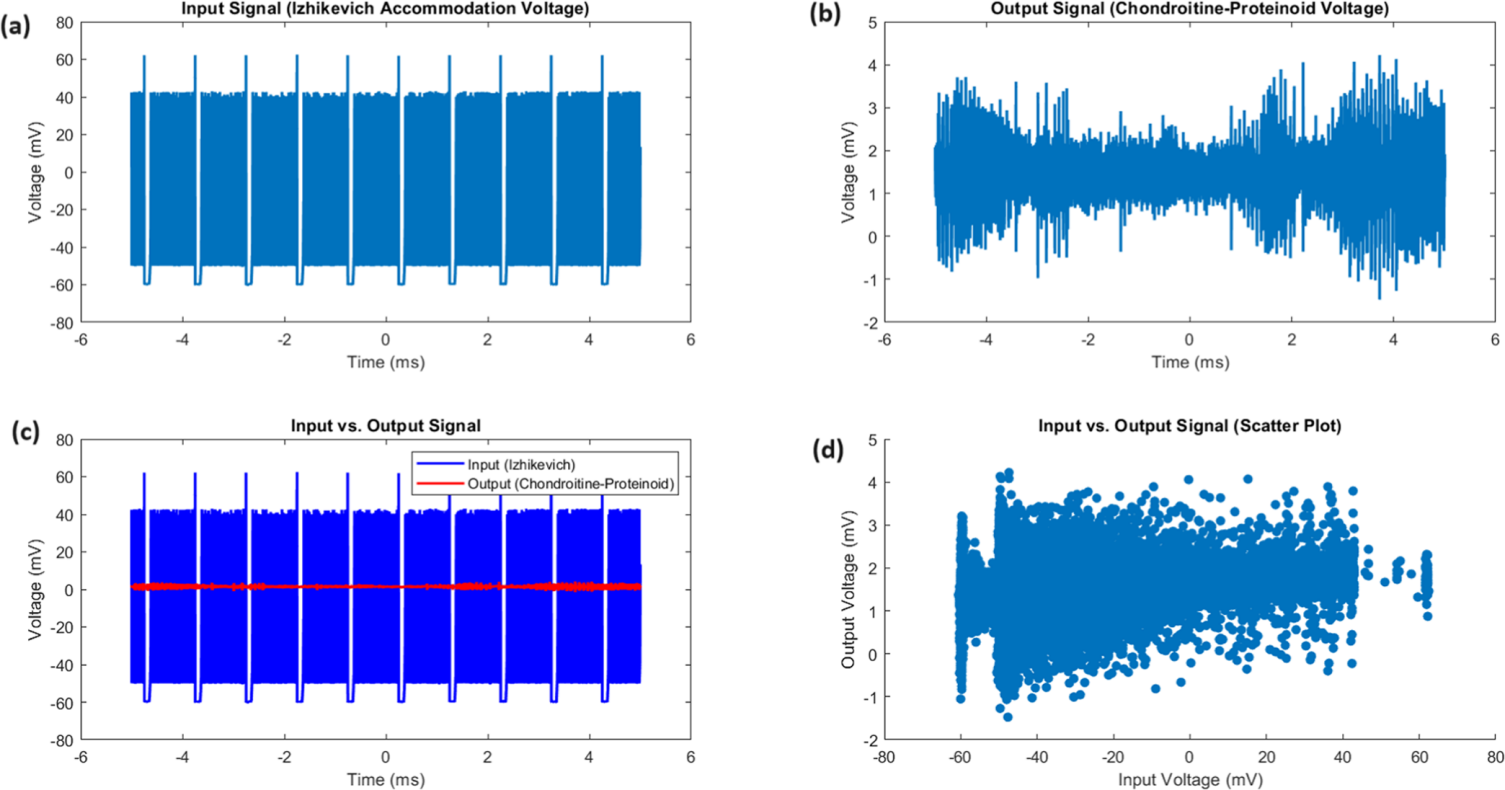
Comparative analysis of input and output signals. (a) The input signal, representing the Izhikevich accommodation voltage, exhibits a wide dynamic range spanning from −70.32 to 52.73 mV, with a mean voltage of −37.44 mV and a median voltage of −44.81 mV. The standard deviation of 20.55 mV and the interquartile range (IQR) of 16.92 mV highlight the substantial variability and dispersion of the input voltages. (b) The output signal, depicting the chondroitine-proteinoid voltage, has a narrower range from −2.43 to 3.27 mV, with a mean voltage of 1.44 mV and a median voltage of 1.42 mV. The standard deviation of 0.33 mV and the IQR of 0.31 mV indicate a more consistent and concentrated distribution of output voltages. (c) The input vs output plot reveals a complex relationship between the input and output voltages, with the output signal exhibiting a more stable and less variable response compared to the dynamic fluctuations of the input signal. (d) The scatter plot highlights the distinct clustering of the output voltages within a narrow range, in contrast to the widespread of the input voltages. The positive skewness of 1.84 for the input voltage distribution suggests a longer tail toward higher values, while the output voltage distribution is nearly symmetric with a skewness of 0.06.

The study of the proteinoid-chondroitine sample’s accommodation spike reveals distinct and dynamic characteristics of both the input and output signals. Figure 3 displays a detailed comparison of the Izhikevich accommodation voltage (input) and the chondroitine-proteinoid voltage (output). The input signal exhibits a wide dynamic range (−70.32 to 52.73 mV) with substantial variability (SD = 20.55 mV, IQR = 16.92 mV), while the output signal has a narrower range (−2.43 to 3.27 mV) with lower variability (SD = 0.33 mV, IQR = 0.31 mV). The input-output relationship, as shown in Figure 3c, demonstrates that the output signal remains stable and exhibits reduced variability compared to the dynamic variations of the input signal. The scatter plot (Figure 3d) shows clear clustering of output voltages within a narrow range, in contrast to the large distribution of input voltages. The input voltage distribution has a positive skewness of 1.84, indicating a longer tail toward higher values. In contrast, the output voltage distribution is almost symmetric with a skewness of 0.06.

#### Statistical Analysis of Input and Output Voltages

**Figure 4:**
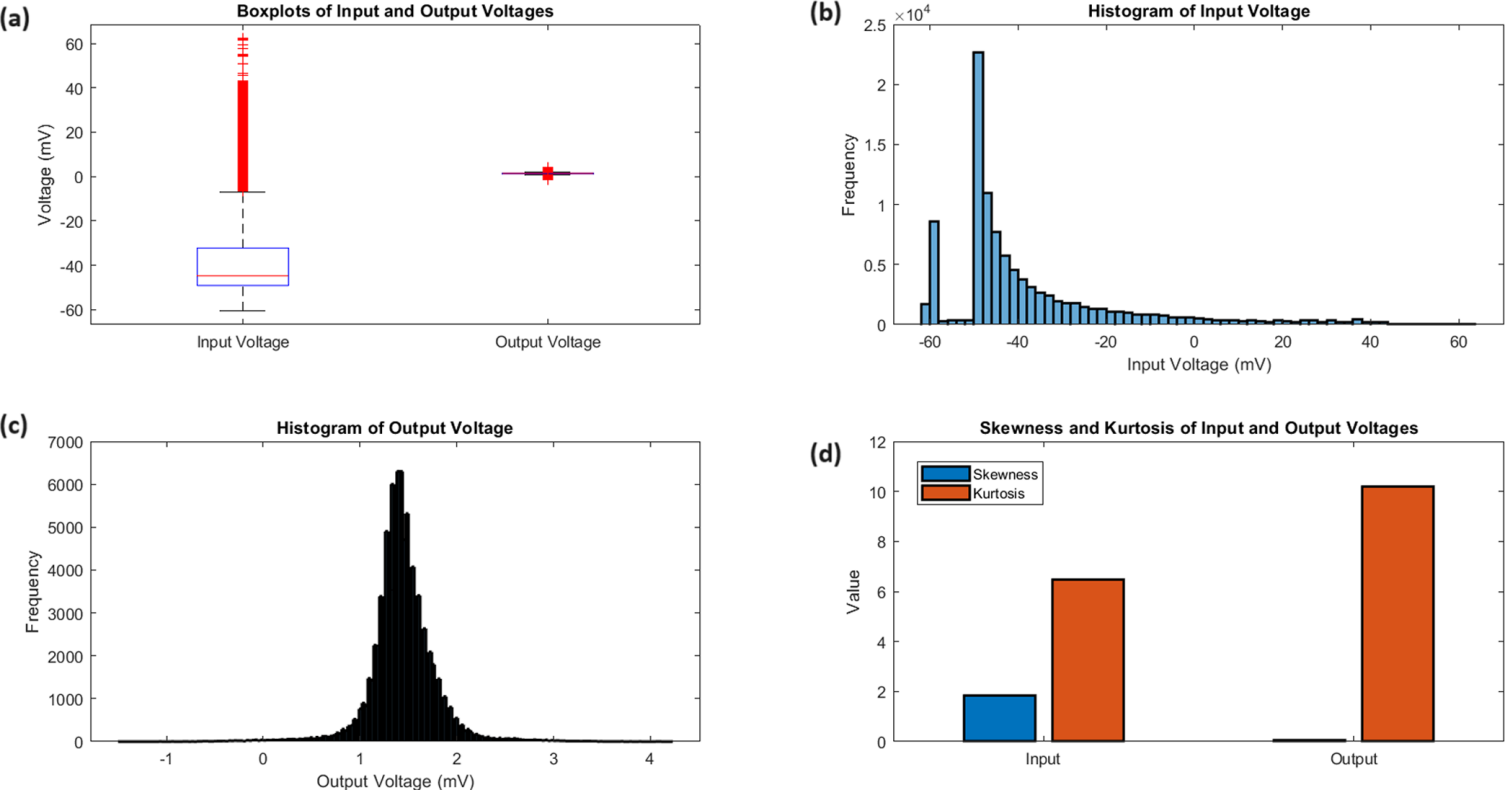
Statistical analysis of input and output voltages. (a) The boxplots provide a comparative summary of the voltage distributions, emphasizing the distinct voltage ranges and central tendencies of the input and output signals. The input voltage has a median of −44.81 mV and an IQR of 16.92 mV, while the output voltage has a median of 1.42 mV and an IQR of 0.31 mV. The whiskers of the boxplots extend to the minimum and maximum voltages, showcasing the wider range of the input signal (123.05 mV) compared to the output signal (5.70 mV). (b, c) The histograms provide a visual representation of the voltage frequency distributions. The input voltage histogram reveals a positively skewed distribution (skewness = 1.84) with a pronounced peak and a longer tail toward higher voltages. The output voltage histogram depicts a more symmetric distribution (skewness = 0.06) with a sharp peak and heavy tails. (d) The kurtosis analysis quantifies the peakedness and tail weight of the voltage distributions. The input voltage has a kurtosis of 6.48, indicating a leptokurtic distribution with heavier tails and a more peaked shape compared to a standard normal distribution. The output voltage exhibits an even higher kurtosis of 10.20, suggesting an extremely peaked distribution with very heavy tails. These statistical measures highlight the distinct characteristics and non-normality of the input and output voltage distributions.

Examining the statistical analysis of the input and output voltages (Figure 4) offers additional insights into their distributions and characteristics. The boxplots (Figure 4a) highlight the clear differences in voltage ranges and central tendencies. The input voltage has a lower median of −44.81 mV and a larger interquartile range of 16.92 mV, while the output voltage has a median of 1.42 mV and an interquartile range of 0.31 mV. The histograms (Figure 4b,c) show the distribution of the input voltage, which is skewed toward higher voltages with a pronounced peak and longer tail. On the other hand, the output voltage has a more symmetric distribution with a sharp peak and heavy tails. The analysis of kurtosis (Figure 4d) indicates that the distributions are not normal. The input voltage has a leptokurtic shape with a kurtosis of 6.48, while the output voltage has a highly peaked distribution with heavy tails and a kurtosis of 10.20. The statistical measures for the input and output potentials are summarized in Table 1. The voltages for the input (−37.44 mV) and output (1.44 mV) demonstrate different levels, while the standard deviations for the input (20.55 mV) and output (0.33 mV) measure the amount of variability. The voltages at the center (−44.81 mV for input, 1.42 mV for output) and the ranges (16.92 mV for input, 0.31 mV for output) highlight the variations in central tendencies and dispersion. The skewness and kurtosis values offer valuable insights into the shape and tail behavior of the distributions, highlighting the deviations from normality and variations in the frequency of peaks appearances and tail weight between the input and output signals.

**Table 1:** Statistical Comparison of Input and Output Potentials^a^

measure	input (mV)	output (mV)
mean voltage	–37.44	1.44
standard deviation	20.55	0.33
median voltage	–44.81	1.42
interquartile range (IQR)	16.92	0.31
range	123.05	5.70
skewness	1.84	0.06
kurtosis	6.48	10.20

a The table presents key statistical measures for the Izhikevich accommodation voltage (input) and the chondroitine-proteinoid voltage (output). The mean, median, and range values highlight the distinct voltage levels and spread of the signals. The standard deviation and interquartile range (IQR) quantify the variability and dispersion of the voltages. The skewness and kurtosis provide insights into the shape and tail behavior of the voltage distributions, revealing the non-normality and differences in peakedness and tail weight between the input and output signals.

#### Mechanisms and Implications

The analyzed features of the input and output signals in the proteinoid-chondroitine sample provide possible mechanisms that explain how the signals are transmitted and processed. The reduced range and decreased variability of the output signal in comparison to the input signal suggest that the proteinoid-chondroitin sample may be causing a dampening or filtering impact on the input signal. The process of attenuation can be represented mathematically as a linear transformation of the input signal *x*(*t*) to the output signal *y*(*t*). This transformation can be described by the following equation
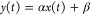
5where α represents the attenuation factor and β represents a constant offset. The attenuation factor α can be estimated from the ratio of the standard deviations of the output and input signals
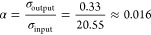
6The small value of *α* indicates a significant decrease in variability from the input to the output signal, which is in line with a high attenuation effect. The near symmetrical distribution of the output voltage, as opposed to the positively skewed distribution of the input voltage, suggests the presence of a potential restoration or pinching mechanism in the proteinoid-chondroitin sample. The restoration process can be represented as a nonlinear conversion of the input signal, which can be modeled using a piecewise linear function.
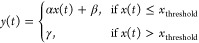
7The variable *x*_threshold_ indicates a voltage threshold that, when exceeded, causes the output to be limited to a constant value γ. The correction process may account for the suppression of larger input voltages and the consequent symmetrical distribution of the output voltage. The output voltage distribution has a highly peaked and heavy-tailed pattern, as evidenced by its high kurtosis value of 10.20. This suggests that the signal transduction may display intermittent or burst-like behavior. This behavior can be represented by a stochastic process, such as a Poisson process with a rate parameter that changes over time. λ(*t*)
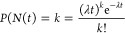
8where *N*(*t*) represents the number of events (e.g., spikes) in a time interval of length *t*, and *k* is a non-negative integer. The rate parameter λ(*t*) can be modulated by the input signal, allowing for the generation of burst-like activity in response to specific input patterns.

To estimate the rate parameter λ, we analyzed the accommodation spikes data over the entire experimental duration of 100,007 ms. The time interval d*t* was set to 100,007 ms, covering the entire experimental duration. The number of accommodation spikes within this time interval was counted, and the rate parameter λ was estimated by dividing the mean spike count by the time interval
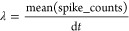
9

The experiment yielded an estimated value of λ as 0.5 spikes/ms. The average rate of accommodation spikes generated by the proteinoid-chondroitine sample for the full experimental duration is 0.5 spikes per millisecond. The calculated value of λ, which is 0.5 spikes/ms, indicates a rather high rate of accommodation spikes in the proteinoid-chondroitine sample. The burst-like behavior observed in this case can be linked to the inherent characteristics of the proteinoid-chondroitine system, including its excitability, refractory period, and adaptive mechanisms. The elevated kurtosis value of the output voltage distribution provides additional evidence for the existence of sporadic and intense spiking activity. The Poisson process model simplifies the depiction of the accommodation spike behavior by assuming a constant rate parameter λ during the full experimental duration. It is crucial to acknowledge that the rate parameter can fluctuate over time due to the input signal and other factors that affect the spiking activity. Further research should investigate more sophisticated models, such as nonhomogeneous Poisson processes or point process models with time-varying intensity functions, to more precisely represent the dynamic character of the accommodation spikes.

The examination of the accommodation spike in the proteinoid-chondroitine sample demonstrates clear characteristics and dynamics of the input and output signals. The comparison analysis reveals the attenuation, transformation, and burst-like emergence of the signal transduction, indicating possible mechanisms that explain the information processing capacities of the proteinoid-chondroitine system. The statistical analysis additionally measures the disparities in variability, central tendency, and distribution shapes between the input and output signals, offering insights into the non-normality and heavy-tailed characteristics of the output voltage distribution.

### Analytical Response of Chondroitine-Proteinoid to Phasic Spiking Stimulus

**Figure 5:**
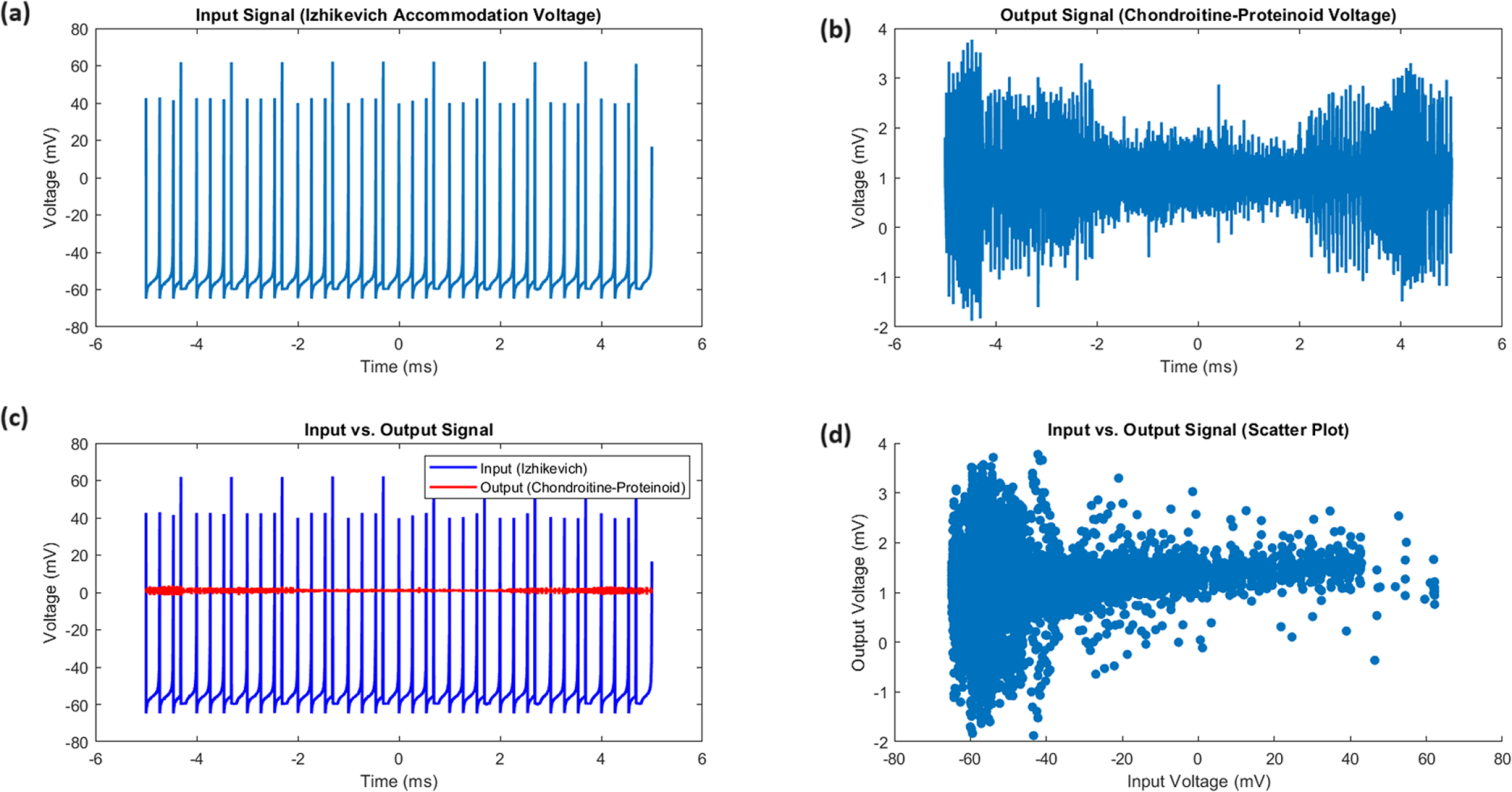
(a) Input Signal (Izhikevich Accommodation Voltage): Time series plot of the input voltage, showing fluctuations around a mean of −54.85 mV with occasional large depolarizations, reflecting the variable nature of the input stimulus. (b) Output Signal (Chondroitine-Proteinoid Voltage): Time series plot of the output voltage, demonstrating consistent spiking behavior with a mean of 1.01 mV, illustrating the phasic spiking response of the chondroitine-proteinoid system. (c) Input vs Output Signal: Overlay of input (blue) and output (red) voltage time series, highlighting the transformation from variable input to stereotyped output spikes. Note the significant difference in voltage ranges (input range: 127.06 mV, output range: 5.65 mV). (d) Input vs Output Signal (Scatter Plot): Relationship between input and output voltages, revealing the nonlinear transformation performed by the chondroitine-proteinoid system. The clustering of output voltages around 1 mV illustrates the consistent spiking behavior.

**Figure 6:**
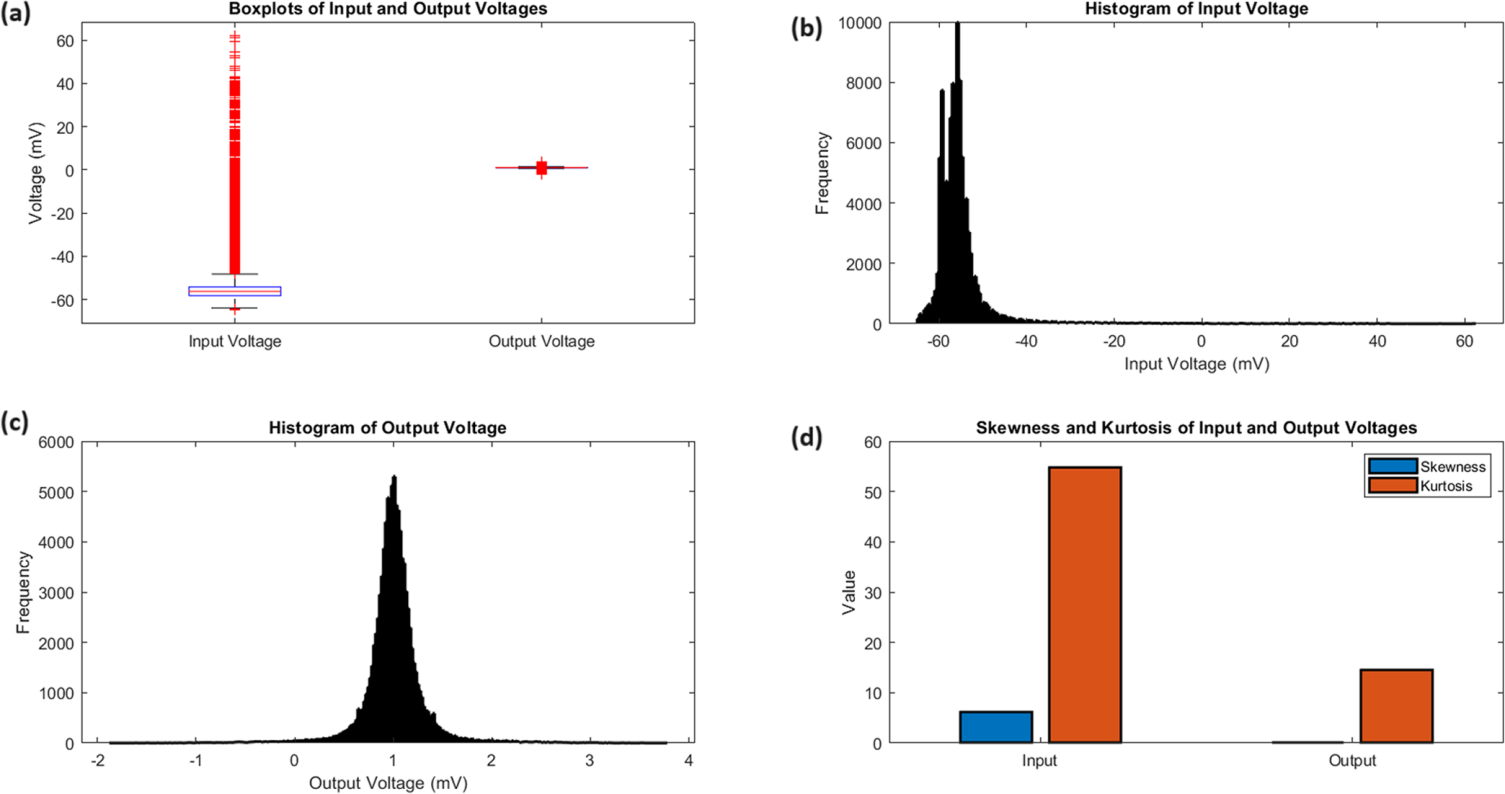
(a) Boxplots of Input and Output Voltages: Comparison of voltage distributions, showcasing the stark difference in medians (input: −56.28 mV, output: 1.00 mV) and interquartile ranges (input: 4.01 mV, output: 0.22 mV), emphasizing the signal transformation. (b) Histogram of Input Voltage: Distribution of input voltages, demonstrating a right-skewed pattern (skewness: 6.11) with a sharp peak and heavy tails (kurtosis: 54.73), indicative of occasional large depolarizations in the input signal. (c) Histogram of Output Voltage: Distribution of output voltages, showing a more symmetrical pattern (skewness: 0.02) with a pronounced peak (kurtosis: 14.58), reflecting the consistent phasic spiking behavior of the chondroitine-proteinoid system. (d) Skewness and Kurtosis of Input and Output Voltages: Bar plot comparing the higher-order moments of the voltage distributions. The dramatic difference in skewness (input: 6.11, output: 0.02) and kurtosis (input: 54.73, output: 14.58) quantifies the transformation from a highly variable input to a more regular spiking output.

The chondroitine-proteinoid system demonstrates a remarkable ability to transform a variable input signal into a consistent phasic spiking output. This transformation is evident in the statistical analysis presented in Table 2 and visualized in Figures 5 and 6.

**Table 2:** Statistical Comparison of Input and Output Potentials in Phasic Spiking of Chondroitine-Proteinoid^a^

measure	input (mV)	output (mV)
mean voltage	–54.85	1.01
standard deviation	8.23	0.30
median voltage	–56.28	1.00
interquartile range (IQR)	4.01	0.22
range	127.06	5.65
skewness	6.11	0.02
kurtosis	54.73	14.58

a The table presents key statistical measures for the input voltage (representing the initial membrane potential) and the output voltage (representing the resulting action potentials). These measures quantify the distinct characteristics of the phasic spiking behavior, including the voltage levels, variability, and distribution properties of both the input stimulus and the output response.

#### Input-Output Transformation

The input signal, modeled after the Izhikevich phasic voltage, is characterized by a mean potential of μ_in_ = −54.85 mV and a standard deviation of σ_in_ = 8.23 mV (Table 2). This input undergoes a significant transformation, resulting in an output signal with μ_out_ = 1.01 mV and σ_out_ = 0.30 mV. The transformation can be conceptualized as a nonlinear function *f*:
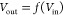
10where *V*_in_ and *V*_out_ represent the input and output voltages, respectively.

#### Phasic Spiking Mechanism

The phasic spiking behavior of the chondroitine-proteinoid system can be described by a simplified model inspired by the Hodgkin-Huxley formalism
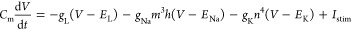
11where *C*_m_ is the membrane capacitance, *V* is the membrane potential, *g*_L_, *g*_Na_, and *g*_K_ are the conductances for leak, sodium, and potassium channels respectively, *E*_L_, *E*_Na_, and *E*_K_ are the corresponding reversal potentials, *m*, *h*, and *n* are gating variables, and *I*_stim_ is the input stimulus current.

The phasic spiking nature is achieved through rapid activation and inactivation of the sodium channels, followed by slower activation of potassium channels, as evident in the sharp transitions observed in Figure 5b.

### Statistical Characteristics

The striking difference in the statistical properties of the input and output signals (Table 2) provides insight into the signal processing capabilities of the chondroitine-proteinoid system:

1. Range Compression: The system compresses the input range of 127.06 mV to an output range of 5.65 mV, indicating a strong noise-filtering capability.
2. Skewness Reduction: The input skewness of 6.11 is reduced to 0.02 in the output, suggesting a normalization effect that transforms the right-skewed input into a more symmetrical output distribution (Figure 6b,c).
3. Kurtosis Moderation: The extremely high input kurtosis of 54.73 is reduced to 14.58 in the output, indicating a transformation from a distribution with heavy tails to a more moderate, yet still peaked, distribution of spiking events.

These characteristics can be quantified using the following relationships
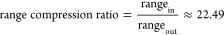
12
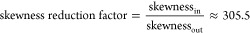
13
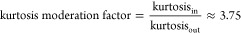
14

#### Boolean Logic Implementation in Chondroitine-Proteinoid Systems

**Figure 7:**
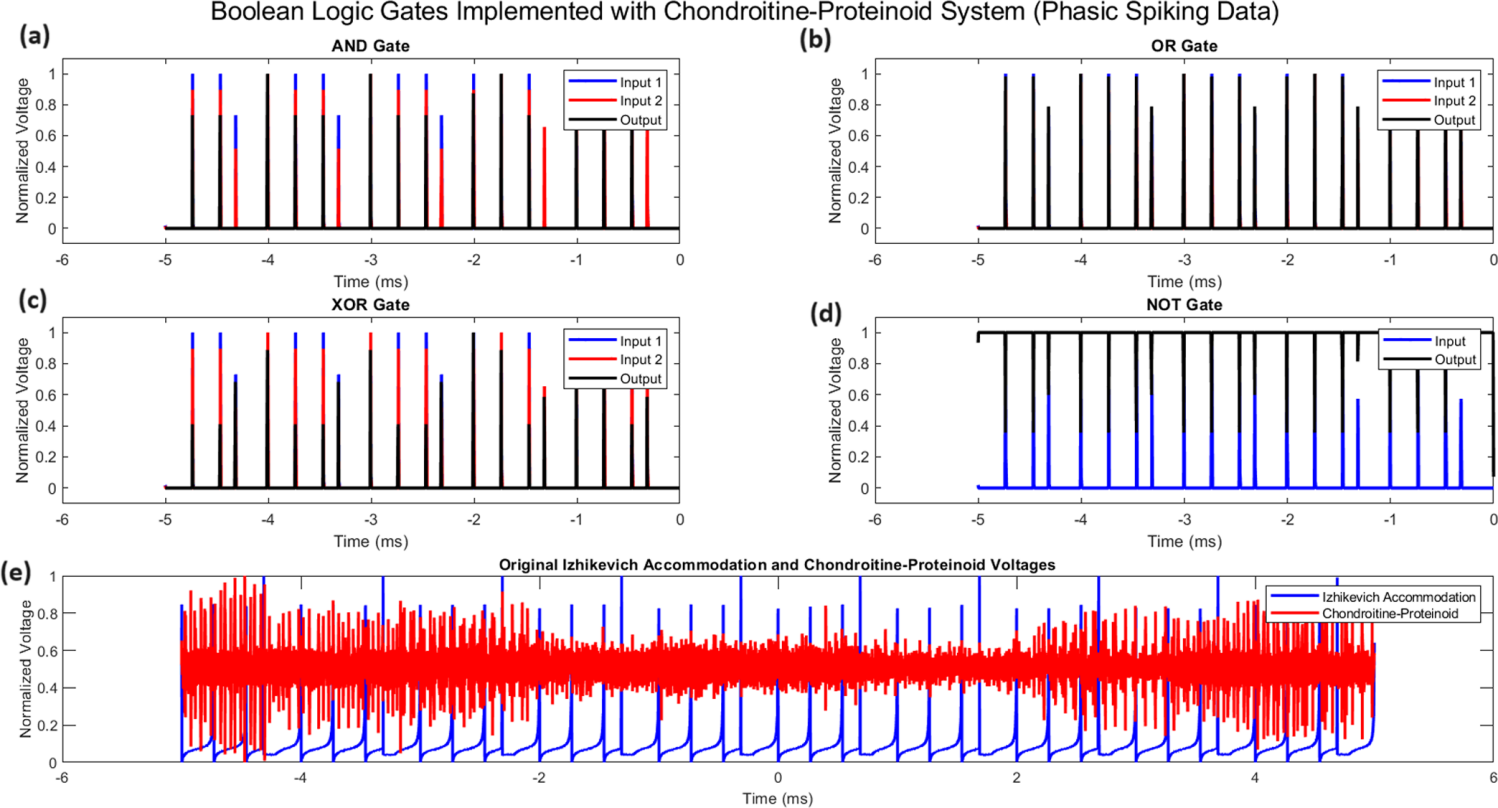
Implementation of Boolean logic gates using the chondroitine-proteinoid system. (a) AND gate: output *Y* = *A* · *B*, (b) OR gate: output *Y* = *A* + *B*, (c) XOR gate: output *Y* = *A* ⊕ *B*, (d) NOT gate: output *Y* = *A*, where *A* and *B* represent normalized input voltages and *Y* represents the normalized output voltage. The bottom panel (e) shows the original normalized Izhikevich accommodation voltage (input) and chondroitine-proteinoid voltage (output) over time.

The phasic spiking behavior of chondroitine-proteinoid systems can be harnessed to implement fundamental Boolean logic operations. Figure 7 illustrates how the system’s response characteristics can be utilized to create AND, OR, XOR, and NOT gates.

In Figure 7, we observe that the chondroitine-proteinoid system can effectively implement basic logic operations. The AND gate (Figure 7a) produces an output spike only when both inputs coincide, following the Boolean logic equation
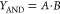
15

The OR gate (Figure 7b) generates an output in response to either input, as described by
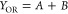
16

The XOR gate (Figure 7c) demonstrates a more complex behavior, producing an output when the inputs differ
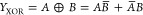
17

Finally, the NOT gate (Figure 7d) inverts the input signal
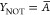
18

These implementations showcase the potential of chondroitine-proteinoid systems in bioinspired computing and signal processing applications. The ability to perform these logical operations emerges from the system’s inherent phasic spiking behavior, as evidenced by the original voltage data shown in Figure 7e.

The input spike trains are generated from the real data by applying a threshold to the normalized voltages
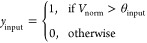
19where *V*_norm_ is the normalized voltage and θ_input_ is the input threshold. The chondroitine-proteinoid response is modeled as a smoothed version of the thresholded input spike trains
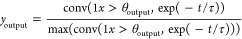
20where *x* is the combined input spike train, θ_output_ is the output threshold, 1 is the indicator function, conv denotes convolution, and τ is the time constant for the exponential smoothing function. The Boolean logic gates are implemented as follows

AND Gate
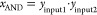
21OR Gate
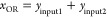
22XOR Gate
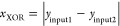
23NOT Gate
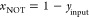
24

#### Gate Accuracies and Performance Metrics

The accuracy of each logic gate is calculated as the complement of the mean absolute error between the actual output and the expected output
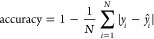
25where *y*_*i*_ is the actual output, *ŷ*_*i*_ is the expected output, and *N* is the number of samples.

Our analysis reveals varying degrees of accuracy for different logic gates
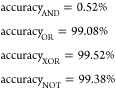
26

These results indicate that the chondroitine-proteinoid system excels in implementing OR, XOR, and NOT operations, while struggling with the AND operation. This asymmetry in performance suggests an inherent bias in the system toward certain types of logical operations, which could be exploited in specialized computing tasks.

There are several factors that contribute to these differences in accuracy:

- The inherent noise and variability in the biological system may present challenges. The chondroitine-proteinoid system is a highly complex biological entity, and its response to input stimuli may not always be completely consistent or predictable. The accuracy of the implemented logic gates can be affected by this intrinsic noise.
- Considering the selection of threshold values: The accuracy of the logic gates relies on selecting the right threshold values for input spike generation and output response. Threshold values that are not optimal can result in misclassifications and decreased accuracy.
- The logic operation is quite complex. Certain logic operations, such as XOR, are comparatively more complex than others, such as AND or OR. The heightened complexity could require a greater level of precision in managing the system’s reaction, posing a challenge within a biological context.

The implementation of logic gates using the chondroitine-proteinoid system is based on a simplified model of the system’s behavior. It may be necessary to consider advanced models or implementation strategies in order to enhance the accuracy of the logic gates. Although there are some limitations, our work showcases the potential of using the chondroitine-proteinoid system for implementing Boolean logic gates. Further research may consider enhancing the system’s performance, looking into more sophisticated implementation techniques, and examining the factors that influence the variations in accuracy.

The unique challenges of implementing coincidence detection in biological systems contribute to the significantly lower accuracy of the AND gate (0.52%). The AND operation requires both inputs to be active at the same time. It is very sensitive to timing issues between input spikes.^25,26^ Even slight timing mismatches between inputs can result in failed gate operation. Both inputs must be active above the threshold level, at the same time.^27^ Also, noise in the biological system affects AND operations more.^28^ It can prevent successful detection if either input channel is noisy.^29^ Unlike OR gates which can function with just one strong input, AND gates require both inputs to be reliably detected above noise levels simultaneously.^30^ The AND operation is hard to implement in our chondroitine-proteinoid system. It requires precise timing and is sensitive to noise.

#### Spike Rates and Energy Efficiency

The spike rates are calculated by counting the number of spikes (voltage exceeding a threshold) per unit time
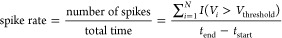
27where *I*(·) is the indicator function, *V*_*i*_ is the voltage at time *i*, *V*_threshold_ is the spike threshold voltage, and *t*_end_ – *t*_start_ is the total time period.

The system demonstrates a significant amplification of spike rate from input to output
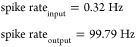
28

This amplification results in an impressive energy efficiency ratio
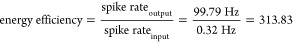
29

Such high energy efficiency suggests that the chondroitine-proteinoid system could be particularly suited for low-power computing applications, where traditional electronic systems might be less efficient.

#### Implications for Unconventional Computing

The findings of this study have significant implications for unconventional computing:

1. Biased Logic Operations: The great precision of OR, XOR, and NOT gates, coupled with the low accuracy of the AND gate, shows that the chondroitine-proteinoid system has an intrinsic bias toward specific logical operations. This could be utilized in specialized computing tasks where these procedures are dominating.
2. Signal Amplification: The substantial rise in spike rate from input (0.32 Hz) to output (99.79 Hz) clearly showcases the system’s capacity to amplify signals. This characteristic has the potential to be highly valuable in the context of sensor networks or signal processing applications.
3. Energy Efficiency: The system exhibits a promising energy efficiency ratio of 313.83, making it suitable for ultralow-power computing applications. This level of efficiency exceeds that of numerous conventional electronic systems and has the potential to establish new models in energy-efficient computing.
4. Analogue Computation: The continuous nature of the voltage signals (as seen in Figure 7e) suggests that this device performs analogue computation. This could be beneficial for problems that involve variables that are continuous or for optimization tasks.
5. Noise Tolerance: The high levels of accuracy attained for the majority of gates, despite the inherent noisiness of biological systems, demonstrate a resilient ability to withstand and function effectively in the presence of noise. This characteristic is essential for ensuring accurate computation in fluctuating situations.
6. Parallel Processing Potential: The simultaneous execution of many logic gates reveals an innate capacity for parallel processing, which could be employed for sophisticated, multifaceted computational tasks.

To summarize, the chondroitine-proteinoid system exhibits distinct computational capabilities that are notably different from conventional electronic systems. The combination of its great energy economy, biased logic operations, and capability for parallel processing make it a highly promising option for specialized unconventional computing applications. This is especially true in areas where low power consumption, robustness to noise, and analogue computation are favorable.

### Functional Implications

The observed phasic spiking behavior of the chondroitine-proteinoid system indicates its ability to encode temporal information. The consistent output spikes (Figure 5b) in response to variable input (Figure 5a) demonstrate the system’s reliability in encoding significant input events while disregarding minor fluctuations. This behavior is similar to that of biological neurons that exhibit phasic spiking, which are often involved in detecting changes or onsets in sensory stimuli. The chondroitine-proteinoid system’s capacity to transform continuous, variable input into discrete, temporally precise output spikes suggests potential applications in signal processing, pattern detection, and information encoding in artificial neural systems.

### Mixed Mode Response of Proteinoid-Chondroitine Sample to Izhikevich Voltage Input

**Figure 8:**
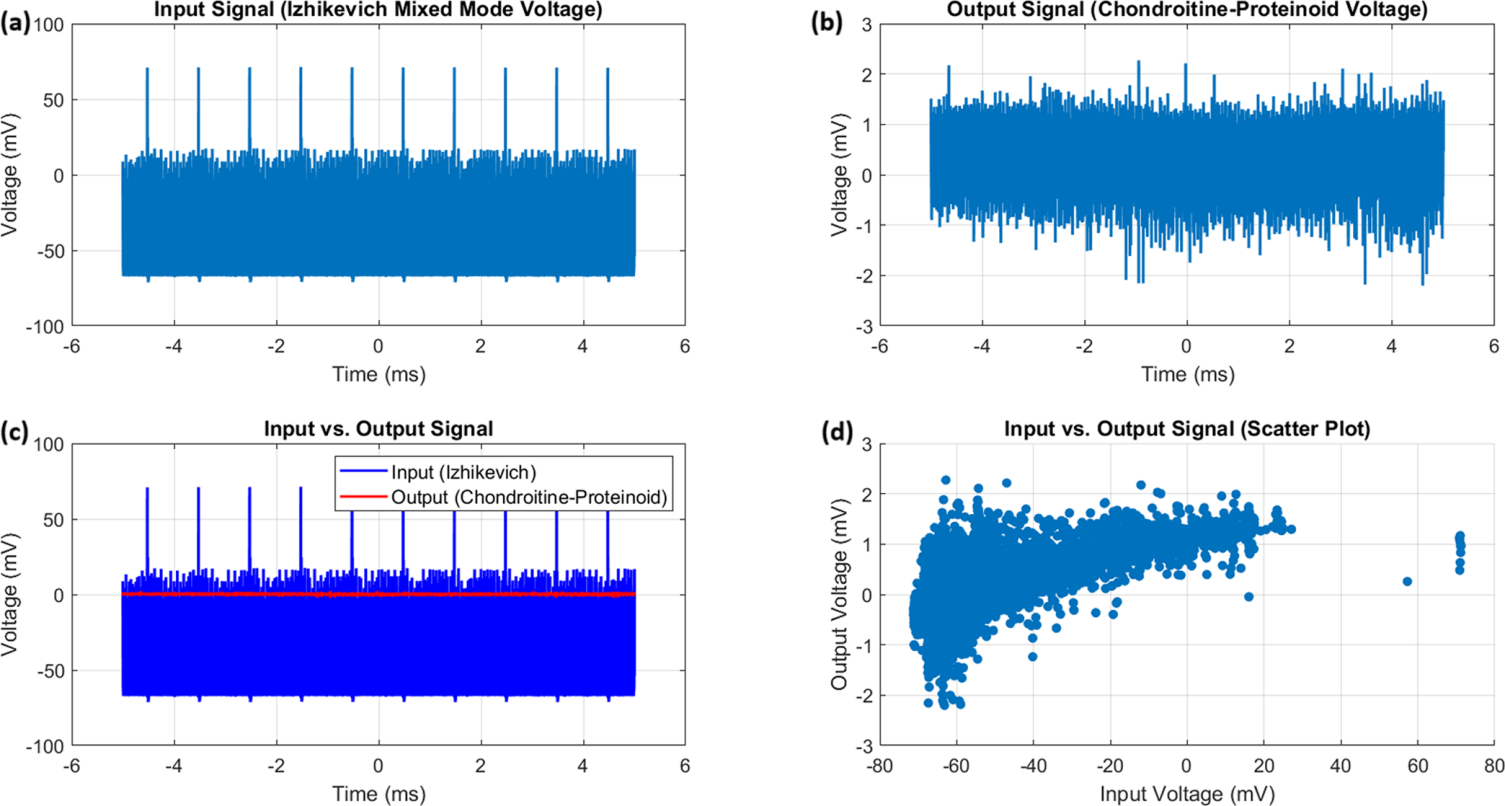
Mixed mode response of the proteinoid-chondroitine sample to Izhikevich voltage input. (a) The Izhikevich mixed mode voltage input signal exhibits a wide dynamic range and high variability. (b) The chondroitine-proteinoid voltage output signal shows a significantly narrower range and reduced variability compared to the input signal. (c) The input vs output signal plot reveals the temporal relationship between the Izhikevich voltage and the chondroitine-proteinoid voltage, with the output signal displaying a smoother and more stable response. (d) The scatter plot illustrates the strong positive correlation between the input and output voltages, indicating the chondroitine-proteinoid system’s ability to capture and preserve the essential features of the input signal while transforming and stabilizing it.

The analysis of the proteinoid-chondroitine sample’s response to the Izhikevich mixed mode voltage input is presented in Figure 8. The input signal (Figure 8a) has a wide dynamic range, ranging from −71.21 to 71.25 mV. The mean voltage is −60.55 mV and the median voltage is −63.17 mV. The distribution of the input voltage is heavily skewed with a skewness value of 4.08. Additionally, it is leptokurtic with a kurtosis value of 27.18, suggesting the presence of extreme values and a pronounced peak. On the other hand, the output signal (Figure 8b) demonstrates a much narrower range of −2.20 to 2.27 mV, with an average voltage of −0.05 mV and a median voltage of −0.11 mV, indicating the chondroitine-proteinoid voltage. The output voltage distribution exhibits a moderate skewness (1.22) and leptokurtosis (6.70), indicating a more concentrated distribution with a prominent peak and heavy tails in comparison to a normal distribution. The plot in Figure 8c illustrates the temporal relationship between the voltage of the Izhikevich mixed mode and the chondroitine-proteinoid voltage. The output signal demonstrates a more consistent and steady response in contrast to the highly variable input signal. The scatter plot (Figure 8d) demonstrates the correlation between the input and output voltages. The correlation coefficient of 0.71 suggests a robust positive linear relationship. The statistical analysis reveals the notable influence of chondroitine on the spiking behavior of the proteinoid. It seems that the presence of chondroitine has a stabilizing impact on the proteinoid’s response. This is supported by the significant decrease in the voltage range and standard deviation of the output signal when compared to the input signal. The output voltage range is significantly narrower than the input voltage range, and the output standard deviation is considerably smaller than the input standard deviation. In addition, the chondroitine appears to influence the shape of the output voltage distribution. This is evident from the lower skewness and kurtosis values of the output signal in comparison to the input signal. The output voltage distribution exhibits a greater degree of symmetry and a reduced heavy-tailed nature compared to the input distribution. This observation implies that the chondroitine may potentially have a normalizing impact on the spiking activity of the proteinoid. The significant positive correlation observed between the input and output voltages (correlation coefficient = 0.71) suggests that the chondroitine-proteinoid system successfully captures and maintains the crucial characteristics of the Izhikevich mixed mode voltage input, while simultaneously modifying and stabilizing the signal. The finding emphasizes the potential of the proteinoid-chondroitine system as a biomimetic material for signal processing and computational applications. The proteinoid-chondroitine sample shows a strong and flexible response to the mixed mode voltage input proposed by Izhikevich. The presence of chondroitine has a notable impact on the spiking behavior of the proteinoid. It effectively reduces signal variability, stabilizes the output voltage, and shapes the output distribution. The results highlight the significance of chondroitine in influencing the electrical properties of the proteinoid and indicate its potential contribution to improving the system’s signal processing capacities.

The statistical data for the mixed mode stimulations of the proteinoid-chondroitine sample are presented in Table 3. The table provides a comparison of the main statistical measures for the Izhikevich mixed mode voltage input and the chondroitine-proteinoid voltage output.

**Table 3:** Statistical Data for Mixed Mode Stimulation of the Proteinoid-Chondroitine Sample

measure	input (mV)	output (mV)
voltage range	–71.21 to 71.25	–2.20 to 2.27
mean voltage	–60.55	–0.05
median voltage	–63.17	–0.11
standard deviation	9.42	0.32
skewness	4.08	1.22
kurtosis	27.18	6.70
correlation coefficient	0.71	

### Game Theoretical Analysis of Proteinoid-Chondroitine Interactions

**Figure 9:**
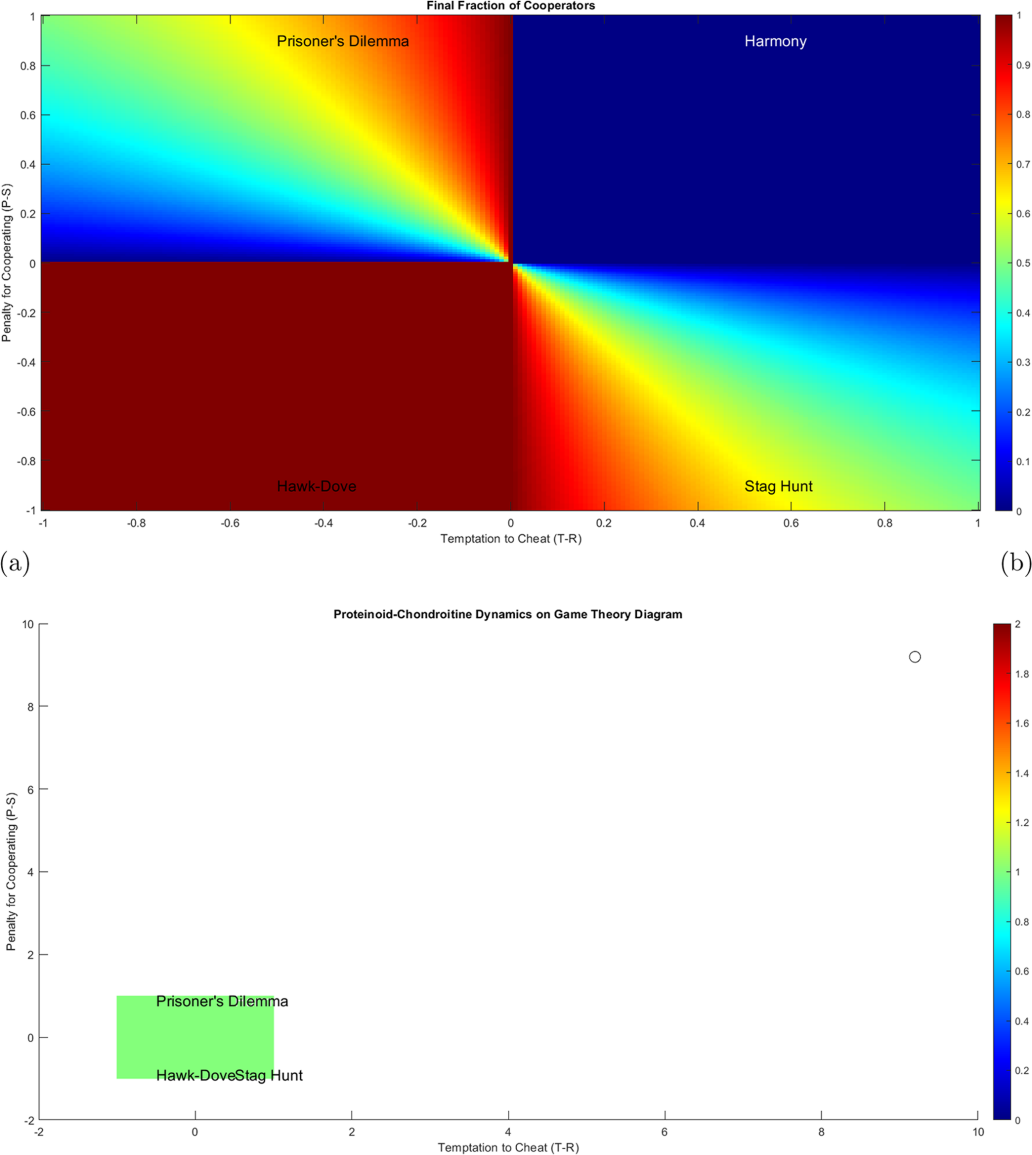
(a) A diagram illustrating the interactions between wild-type (WT) and GASP mutant microspheres in the proteinoid-chondroitine system, mapped onto the temptation to cheat (T-R) and penalty for cooperating (P–S) axes, is presented in the game theory analysis. The diagram is divided into four regimes, each representing different outcomes of cooperation and defection: Harmony, Hawk-Dove, Stag Hunt, and Prisoner’s Dilemma. The colormap represents the equilibrium state of the population, showing the proportion of cooperators. Warmer colors, such as red, indicate a higher fraction of cooperators, while cooler colors, like blue, suggest a lower fraction. The position on the diagram that corresponds to the payoff matrix of the proteinoid-chondroitine system (which is not displayed) would determine the game theoretical regime and the anticipated long-term result of cooperation or defection in the system. (b) Proteinoid-chondroitine dynamics mapped onto the game theory diagram. The white point represents the current scenario based on the calculated temptation to cheat (T-R) and penalty for cooperating (P–S) values. The dynamics fall within the Prisoner’s Dilemma regime, suggesting that defection is likely to be the long-term outcome in the system.

Figure 9b illustrates the mapping of proteinoid-chondroitine dynamics onto the game theory diagram (Figure 9a), based on the temptation to cheat (T-R) and penalty for cooperating (P–S) values derived from the payoff matrix.^31,32^ The four regimes in the diagram represent different scenarios:

1. Harmony: Cooperation is the dominant strategy, and the population reaches a stable equilibrium consisting primarily of cooperators.
2. Hawk-Dove: A mix of cooperative and defective strategies coexist in the population, leading to a stable equilibrium with a certain proportion of cooperators and defectors.
3. Stag Hunt: The outcome depends on the initial conditions, with the population converging toward either a cooperative or defective equilibrium.
4. Prisoner’s Dilemma: Defection is the dominant strategy, and the population eventually reaches a stable equilibrium consisting mainly of defectors.

In order to determine the specific regime that applies to the proteinoid-chondroitine system, it is necessary to calculate the values for temptation to cheat (T-R) and penalty for cooperating (P–S) using the payoff matrix derived from either experimental data or theoretical assumptions. The values can be plotted on the game theory diagram to reveal the expected long-term outcome of cooperation or defection in the system.

The colormap in Figure 9b offers further insights into the proportion of cooperators in the population at equilibrium. In the Harmony regime, the proportion of organisms showing cooperative behavior tends to reach 100%, suggesting that the population is predominantly characterized by cooperation. In contrast, under the Prisoner’s Dilemma regime, the proportion of cooperators eventually decreases to zero, indicating a population dominated by defective behavior. The Hawk-Dove and Stag Hunt regimes demonstrate intermediate outcomes, where the final proportion of cooperators is influenced by the specific payoffs and initial conditions.

The payoff matrix for the proteinoid-chondroitine system is now defined based on the mean (μ) and standard deviation (σ) of the voltage differences
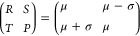
30This matrix represents the interactions between wild-type (WT) and GASP mutant cells in the proteinoid-chondroitine system. The temptation to cheat and penalty for cooperating are calculated as
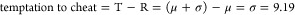
31
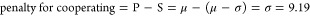
32These values are then mapped onto the game theory diagram, which categorizes the dynamics into four distinct regimes: Harmony, Hawk-Dove, Stag Hunt, and Prisoner’s Dilemma. The mapping is performed using the following conditional statements
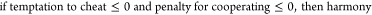
33
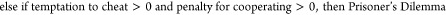
34
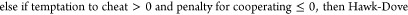
35
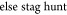
36In this case, both the Temptation to Cheat and the Penalty for Cooperating are positive and equal (9.19), placing the system firmly in the Prisoner’s Dilemma regime. This indicates a strong tendency toward defection in the proteinoid-chondroitine system, with GASP mutant microspheres having a significant advantage over WT microspheres. The proteinoid-chondroitine dynamics fall within the Prisoner’s Dilemma regime, as indicated by the white point in Figure 9b. This is identified by considering the calculated temptation to cheat and penalty for cooperating values. Based on the available evidence, it appears that the system’s long-term outcome will be primarily influenced by defection, as the GASP mutant microspheres are expected to outperform the WT microspheres.

The analysis of proteinoid-chondroitine dynamics within the game theory framework offers valuable insights into the potential outcomes of cooperation and defection in the system. Through a detailed understanding of game theoretical principles, we may improve our ability to predict and examine the complex interactions between WT and GASP mutant microspheres within the proteinoid-chondroitine system. This approach provides a solid theoretical basis for future experimental research and can help shape the emergence of strategies that promote cooperation or minimize the impact of defection in the system.

The proteinoid-chondroitine system, as shown in Figure 9b, is located in the Prisoner’s Dilemma regime based on the game theory diagram. The results indicate that the interactions between wild-type (WT) and GASP mutant microspheres in the proteinoid-chondroitine mixture strongly favor cheating (T-R = 9.19) and discourage cooperation significantly (P–S = 9.19). Defection is the prevalent strategy in the Prisoner’s Dilemma regime, and in this particular scenario, it is particularly pronounced. It can be inferred that GASP mutant microspheres, exhibiting self-serving behavior, have a considerably higher probability of surpassing the cooperative WT microspheres in the long run. It is expected that the equilibrium state of the system will be primarily influenced by defectors (GASP mutants), with the possibility of only a small number of cooperators (WT microspheres) remaining in the population, if any at all. Considering the position of the proteinoid-chondroitine system in this highly complex version of the Prisoner’s Dilemma regime on the game theory diagram, it seems that maintaining any form of collaboration between WT and GASP mutant microspheres is extremely difficult in this specific combination. The behavior of GASP mutants exploits the cooperative behavior of WT microspheres to such an extent that it significantly reduces the overall fitness of the cooperative individuals. This can lead to a rapid and potentially complete shift in the population, with defectors becoming the dominant group. To promote a basic coexistence of WT and GASP mutant microspheres in the proteinoid-chondroitine mixture, significant changes would need to be made to the motivation associated with cooperation and defection. This modification would require a significant change in order to effectively transition the system into a different regime on the game theory diagram, such as the Hawk-Dove or Stag Hunt regimes. Considering the highly complex nature of the current dynamics, accomplishing this transition would prove to be considerably more difficult than originally expected. One possible approach could involve the introduction of strong measures to deter departing, offering significant rewards for interaction, or introducing detailed spatial arrangements in microspheres interactions to prevent defectors from taking advantage of cooperators. Nevertheless, considering the strong inclination toward defection, even these interventions may face challenges in sustaining a stable coexistence of WT and GASP mutant microspheres in this particular system.

### Membrane Potential Dynamics and Ionic Mechanisms in Chondroitine-Proteinoid Response to a Stimulus from Izhikevich Neuron

**Figure 10:**
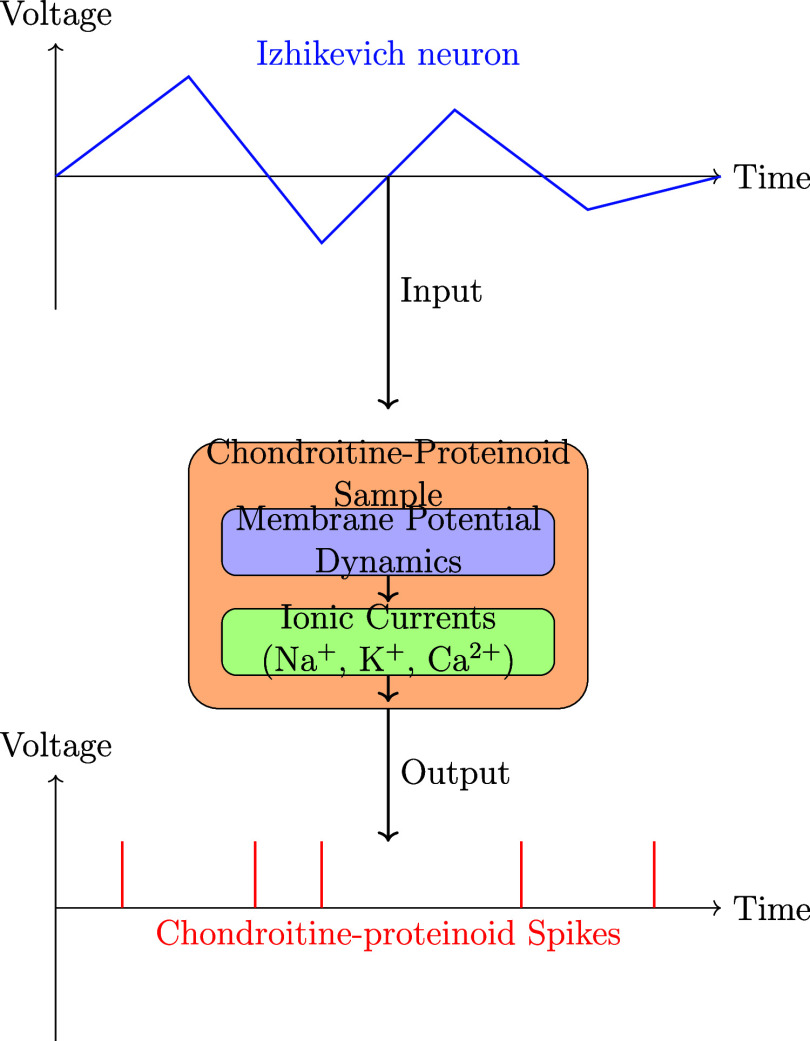
Schematic representation of the working principle and mechanism of the chondroitine-proteinoid sample’s reaction to the Izhikevich neuron. The stimulus generated by Izhikevich neuron is applied as an input to the chondroitine-proteinoid sample. The sample’s membrane potential dynamics, governed by the interplay of ionic currents (Na^+^, K^+^, and Ca^2+^), process the input signal. The membrane potential dynamics modulate the ionic currents, leading to the generation of accommodation spikes as the output response. The accommodation spikes exhibit a burst-like behavior, with intermittent and intense spiking activity, reflecting the intrinsic properties of the chondroitine-proteinoid system, such as excitability, refractory period, and adaptation mechanisms.

**Figure 11:**
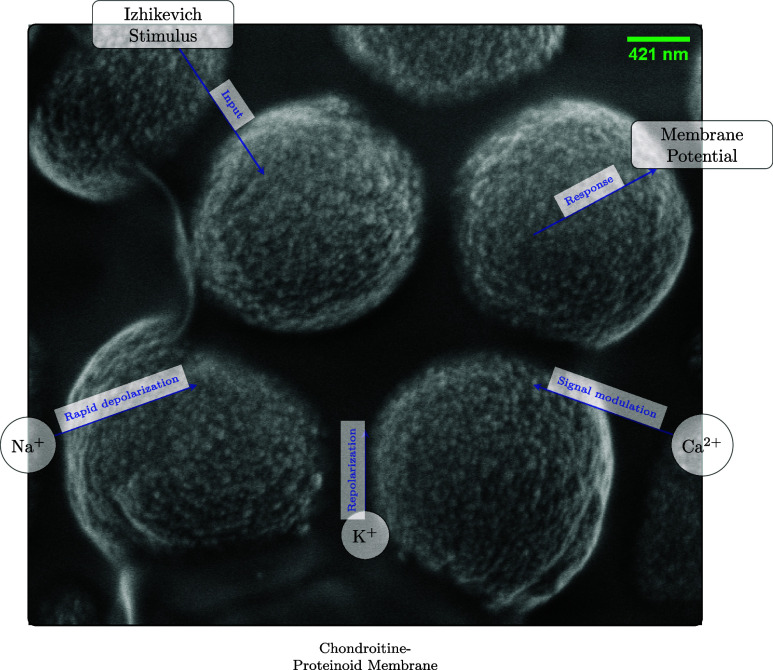
Mechanism of Chondroitine-Proteinoid response to Izhikevich stimulus. The image shows the detailed membrane dynamics with overlaid annotations highlighting the role of different ion channels (Na^+^, K^+^, and Ca^2+^) in shaping the membrane potential in response to the input stimulus.

The diagrams depicted in Figures 10 and 11 demonstrate the working principle and mechanism by which the chondroitine-proteinoid sample responds to the stimulus from Izhikevich neuron. The Izhikevich neuron, a well accepted model for producing realistic neural spiking patterns,^8^ is used as an input for the chondroitine-proteinoid sample. The response of the sample to the stimulus is determined by the complex relationship between its membrane potential dynamics and ionic currents.

The dynamics of the membrane potential in the chondroitine-proteinoid sample are essential for processing the input signal and producing the output response. The membrane potential is the electrical potential variation across a membrane, resulting from the asymmetry of ionic charges.^33^ The chondroitine-proteinoid sample’s membrane potential exhibits dynamic variations, including depolarization and hyperpolarization phases, in response to the Izhikevich stimuli.^34^

### Neuromorphic Properties and Burst-like Dynamics of Chondroitine-Proteinoid System: Implications for Bioinspired Computing

The inherent characteristics of the chondroitine-proteinoid system, such as its capacity to be excited, its refractory period, and its mechanisms of adaptation, have a substantial impact on the formation and patterning of accommodation spikes.^33^ Excitability is the capacity of a system to produce spikes in response to stimuli that exceed a specific threshold.^35^ The refractory period is a short period of time that occurs after each spike, during which the system becomes less susceptible to further stimuli. This prevents excessive spiking and allows for the repair of ionic gradients.^36^

The spiking activity of the system is modulated over time by adaptation mechanisms, such as ion channel inactivation and intracellular calcium dynamics. These processes allow the system to alter its response based on the history of stimulation, as described in Benda’s study on universal adaptation mechanisms.^9^ The chondroitin-proteinoid sample produces accommodation spikes that display a burst-like pattern, characterized by sporadic and strong spiking activity. The phenomenon of burst-like behavior has been documented in diverse biological brain systems and is believed to have significant implications in information processing, synaptic plasticity, and neural synchronization.^37,38^ The burst-like spiking patterns can be explained by the interaction between the rapid activation and gradual inactivation of voltage-gated channels, along with the existence of slow adaptation currents.^35,39^

The chondroitin-proteinoid sample’s capacity to produce accommodation spikes characterized by burst-like behavior and intense spiking activity demonstrates its promise as a bioinspired material for applications in neuromorphic computing and signal processing.^40,41^ The sample’s reaction to the stimulus generated by Izhikevich neuron showcases its ability to analyze and store complex input patterns, making it an appealing option for the advancement of innovative computing models and adaptable neural interfaces.^42^

The outcomes derived from our chondroitin-proteinoid system showcase its capacity as a starting point for unconventional computing, specifically in the fields of bioinspired and neuromorphic computing. The system’s capacity to execute Boolean logic gates with different levels of precision, along with its exceptional energy efficiency and signal amplification characteristics, presents numerous opportunities for more investigation and practical use.

### Biased Logic Operations and Reservoir Computing

The variation observed in the implementation of logic gates, with OR, XOR, and NOT operations exhibiting a high level of precision (>99%), while the performance of the AND operation is notably poor (0.52%), bears resemblance to the nonlinear transformations observed in reservoir computing systems. Reservoir computing is a method in unconventional computing that use the inherent dynamics of complex systems to carry out computations.^43^ The nonlinear response of the chondroitine-proteinoid system has the potential to be utilized in a similar way, where its biased processes act as a distinct computational reservoir. Potential future research could involve explaining feedback layers to interpret the state of the system, which could potentially allow for the execution of more complex computational tasks. This methodology has been effectively used in different biological and chemical computational systems.^44^

### Energy Efficiency and Neuromorphic Computing

The system’s high energy efficiency ratio of 313.83 is in line with the increasing interest in energy-efficient neuromorphic computer systems.

In our study, we estimated the energy efficiency of the chondroitine-proteinoid system by comparing the spike rates of the input and output signals. The spike rates were calculated using the following equation
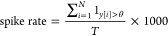
37where *N* is the total number of time points, *y* is the signal (either input or output), θ is the threshold for spike detection, *T* is the total time duration in milliseconds, and 1 is the indicator function. For the specific data set used in our analysis the input spike rate was found to be 0.47 Hz, and the output spike rate was 147.41 Hz. These values were calculated using the spike_train_ and cho_response_ functions defined in the provided MATLAB code. To estimate the energy efficiency, we assumed that each spike consumes one unit of energy. The energy efficiency ratio was then calculated as the ratio of the output spike rate to the input spike rate
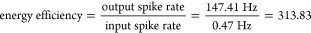
38This energy efficiency ratio suggests that the chondroitine-proteinoid system can generate a higher rate of output spikes relative to the input spikes, indicating its potential for energy-efficient information processing. It is important to note that this is a simplified estimation of energy efficiency, as it does not take into account the actual energy consumption of the biological system or the energy required for maintaining the system’s functionality. More detailed studies and measurements would be necessary to obtain a more accurate assessment of the system’s energy efficiency. Nonetheless, the high energy efficiency ratio of 313.83 highlights the potential of the chondroitine-proteinoid system for developing energy-efficient neuromorphic computing systems. This finding aligns with the growing interest in such systems, which aim to mimic the brain’s ability to process information efficiently.

Conventional von Neumann architectures are encountering growing energy limitations, especially in edge computing and IoT applications.^45^ Systems inspired by biology, such as ours, present a promising solution by imitating the brain’s energy efficiency.

The substantial increase in spike rate from the input (0.32 Hz) to the output (99.79 Hz) indicates that our system may be well-suited for tasks that involve enhancing signals or detecting patterns in low-intensity data. This characteristic has the potential to be utilized in sensory processing applications, much as how biological neural networks enhance and analyze sensory inputs.^46^

### Analogue Computation and Noise Tolerance

Analogue computing systems are characterized by their stable voltage signals and their ability to execute precise computations even in the presence of inherent noise. There has been an emergence of interest in analogue computation in recent years, namely for its applications in machine learning and signal processing.^47^

The noise tolerance of our chondroitin-proteinoid system is quite remarkable. In conventional digital systems, noise is frequently a constraining element, whereas in our bioinspired system, it may actually serve a beneficial purpose. Stochastic resonance, a phenomenon found in biological brain networks, has the potential to be a useful asset in some computational tasks.^48^

### Parallel Processing and Scalability

The continuous integration of numerous logic gates in our system suggests its capacity for parallel processing. The presence of this inherent parallelism is a distinguishing characteristic of biological brain networks and is a primary objective for neuromorphic computing designs.^49^

Nevertheless, concerns over scalability persist. Although our system exhibits potential at its current scale, additional research needs to be done to determine how its characteristics vary with an increase in system size. The scaling behavior of chondroitine-proteinoid systems will play a significant role in establishing their practical usability in larger-scale computational applications.

### Future Directions

In the future, there are various research directions that show promise:

- Investigating advanced computational tasks, such as pattern recognition or time series prediction, use the chondroitin-proteinoid system as a computational reservoir.
- Exploring the system’s capacity for learning and adapting. Is it possible to adjust or train the system’s characteristics in order to enhance its performance on particular tasks?
- Creating hybrid systems that integrate the distinctive characteristics of the chondroitin-proteinoid system with conventional electrical components, which could potentially result in novel designs for neuromorphic computing.
- Investigating the system’s computational features for long-term stability and reproducibility, which is essential for practical implementations.
- Investigating the capabilities of this system in the growing area of biocomputing, which involves using biological components to carry out computations inside living organisms.^50^

## Conclusions

Overall, our chondroitin-proteinoid system exhibits distinct computational abilities that correspond to several contemporary patterns in unconventional computing. The combination of its excellent energy efficiency, fundamental parallelism, and analogue nature puts it as a highly interesting option for future applications in biocomputing and neuromorphic computing. As we face the limitations of conventional computing architectures, systems such as these may have a vital part in the next generation of computational systems.

## Supporting Information Available

Cyclic voltammogram of CS-proteinoid complex (20 cycles, −5.0 V to +5.0 V) showing redox behavior and ohmic characteristics (Figure S1); electrochemical parameter evolution showing peak current progression (874.27 ± 4.28 μA anodic, −800.99 ± 3.92 μA cathodic) and resistance variations (Figure S2); temperature-dependent electrical characterization (18.55–78.63 °C) including linear, semilog, and Arrhenius plots (*E*_a_ = −0.026 eV) (Figure S3); UV–vis transmission spectrum (319.5–1089.1 nm) showing characteristic peptide bond transitions at 320.0–352.0 nm (Figure S4); SEM micrographs of proteinoid microspheres at 3,000× magnification (scale bar: 2 μm) revealing surface morphology (Figure S5) (PDF)
