# Supplementary material for: Chondroitin Sulfate and Proteinoids in Neuron Models
Source: ACS Appl Bio Mater. 2025 Jan 8;8(1):854–69. doi: 10.1021/acsabm.4c01678 (PMC11752506; doi:10.1021/acsabm.4c01678)
Supplement: Supplementary file 1 — mt4c01678_si_001.pdf [file mt4c01678_si_001.pdf]

Supporting Information

Chondroitin Sulphate and Proteinoids in Neuron Models

Panagiotis Mougkogiannis[1,*] and Andrew Adamatzky[1]

[1]Unconventional Computing Laboratory, University of the West of England, Bristol, UK

Email: Panagiotis.Mougkogiannis@uwe.ac.uk

1 Electrical Property Characterization of CS-Proteinoid Solutions

We used cyclic voltammetry to test the CS-protein complex solution's electrical properties. Figure S1 shows the cyclic voltammogram from 20 cycles between -5.0 V and $+5.0$ V. It has quasi-linear regions at high positive and negative potentials. This indicates ohmic behavior in these ranges. At near 0 V, nonlinear regions are seen. They suggest complex charge transfer processes at the electrode-solution interface. The system is very stable across all cycles. The electrochemical response drifted minimally. Its relative standard deviation (RSD) is just 0.49%. Further analysis of the electrochemical parameters is presented in Figure S2. The peak current evolution (Figure S2a) reveals stable anodic and cathodic responses after initial equilibration. The anodic current stabilizes at 874.27 ± 4.28 μA. The cathodic current is -800.99 ± 3.92 μA. The peak current ratio (I_a/I_c) of 1.09 suggests good reversibility of the electron transfer process. The resistance evolution (Figure S2b) shows periodic fluctuations with a mean value of 4.13×10^{-5} kΩ and an RSD of 2.02%. Notable local maxima in resistance occur at cycles 4, 12, and 20, indicating cyclical variations in the electrode-solution interface properties. The high peak potential separation (ΔE_p) of 10.016 V indicates considerable electron transfer resistance in the system. However, it remains stable throughout the measurement series.

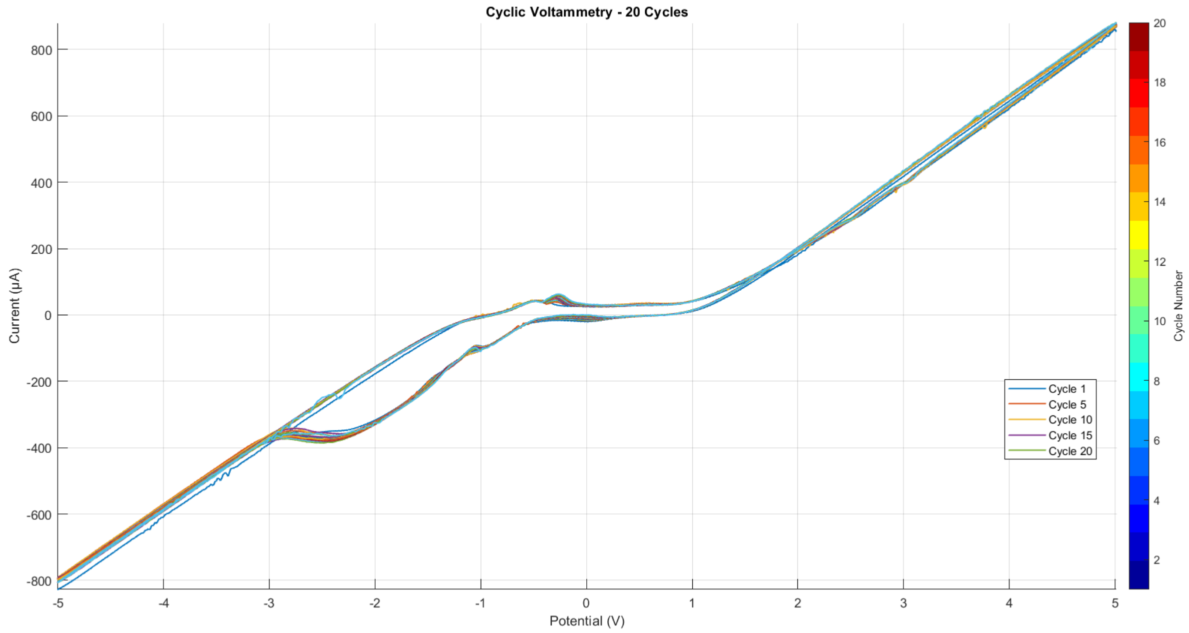

Figure S1: Cyclic voltammogram of CS-protein complex solution measured over 20 consecutive cycles from -5.0 V to $+5.0$ V at room temperature. The current response ranges from -800 μA to $+800$ μA, exhibiting characteristic redox behaviour. The system shows notable stability across cycles (RSD $=$ 0.49%) with a peak current ratio ($I_{\mathrm{a}}/I_{\mathrm{c}}$) of 1.09. A peak potential separation (ΔE_{p}) of 10.016 V indicates significant electron transfer resistance. The colour gradient shows cycle progression from 1 (blue) to 20 (red). It demonstrates excellent reproducibility with minimal drift in the electrochemical response. The quasi-linear regions at high positive and negative potentials suggest ohmic behaviour. The nonlinear regions near 0 V indicate complex charge transfer processes at the electrode-solution interface.

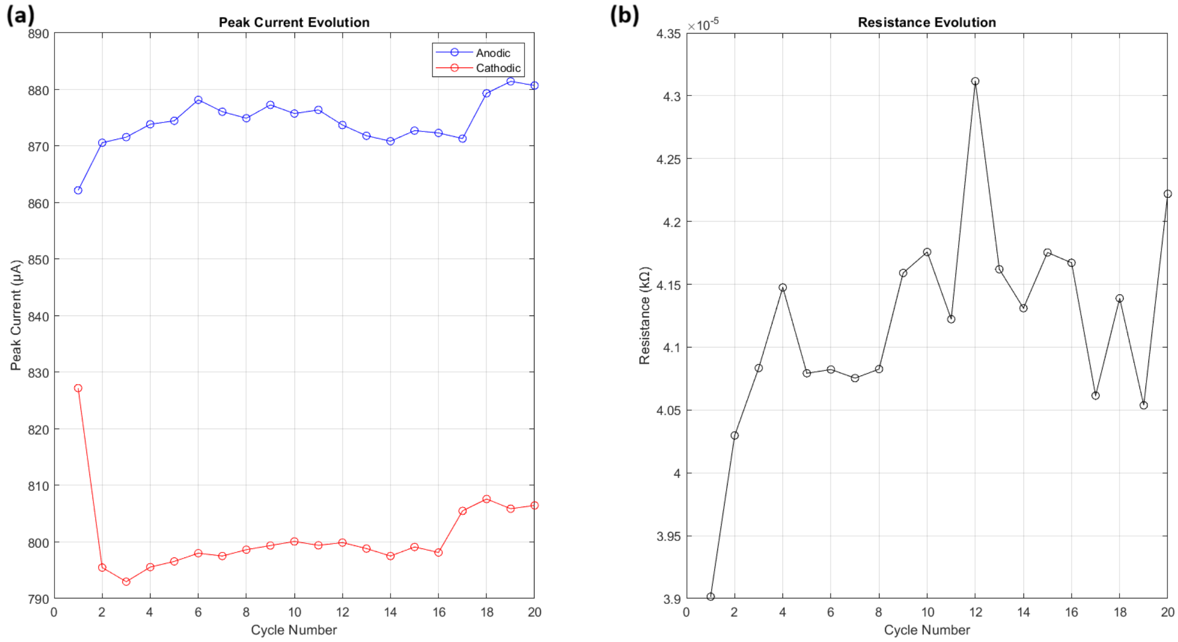

Figure S2: Evolution of electrochemical parameters over 20 cycles for the CS-protein complex solution. (a) Peak current progression showing anodic (blue circles, 874.27 ± 4.28 μA) and cathodic (red circles, -800.99 ± 3.92 μA) currents. The system stabilizes after initial cycles. Anodic currents ranged from 862 to 880 μA. Cathodic currents ranged from -793 to -827 μA. (b) Cycle-dependent resistance varied with a mean of 4.13×10^{-5} kΩ and a 2.02% standard deviation. The resistance profile has local maxima at cycles 4, 12, and 20. This suggests periodic fluctuations in the electrode-solution interface properties. Both metrics demonstrate good electrochemical stability with minor variations across successive cycles.

2 Temperature-Dependent Electrochemical Characterization

We measured the resistance of the CS-Proteinoid complex at 18.55-78.63 °C to test its temperature-dependent electrical properties, as shown in Figure S3. The resistance exhibits a strong temperature dependence, decreasing monotonically from 1.805 kΩ to 0.283 kΩ over the measured range (Figure S3a). This represents an 84.3% reduction in resistance, indicating significant thermal sensitivity of the material. The semi-log plot (Figure S3b) shows an exponential decay of resistance with increasing temperature. This is typical of semiconductor-like materials.

Arrhenius analysis (Figure S3c) shows excellent linearity ($R^2 = 0.9982$) across the entire temperature range, suggesting a single dominant conduction mechanism. The calculated activation energy (E_a) is -0.026 eV, with a pre-exponential factor of 3.215×10^{-5} kΩ. The negative activation energy means that resistance drops as temperature rises. This is typical of semiconductors. The resistance shows key transitions at 41.20 °C and 65.55 °C. At these points, the resistance dropped to 0.760 kΩ (42.1% of the initial value) and 0.379 kΩ (21.0%), respectively. This suggests possible changes in the CS-Proteinoid complex at these temperatures.

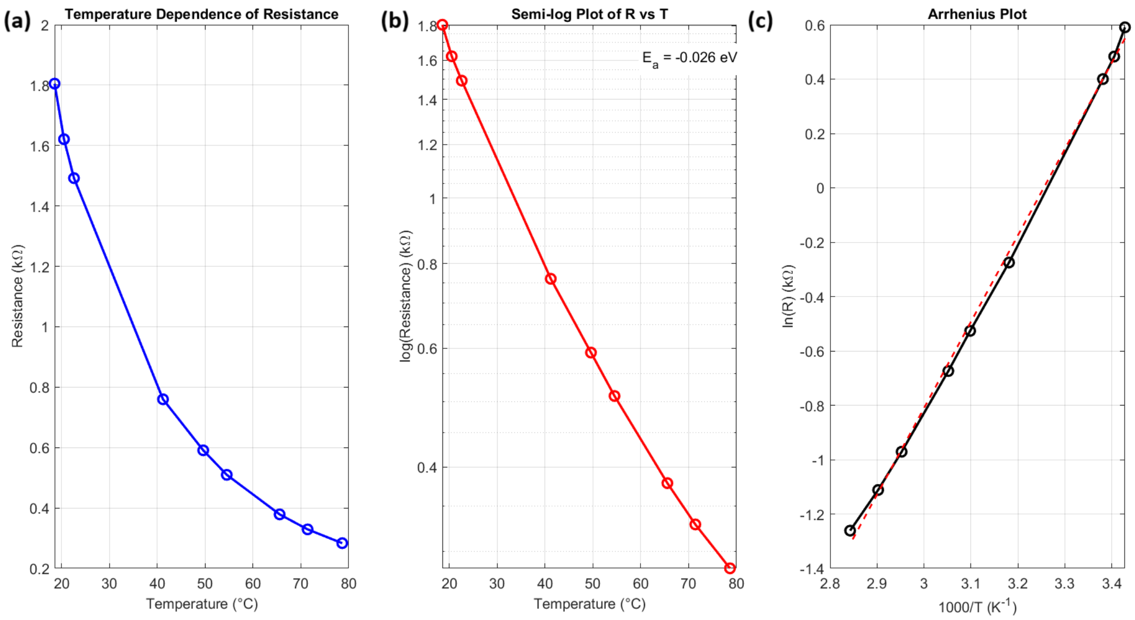

Figure S3: Temperature-dependent electrical characterization of CS-Proteinoid complex. (a) A linear plot of resistance vs. temperature shows a monotonic decrease from 1.805 kΩ at 18.55 °C to 0.283 kΩ at 78.63 °C. This is an 84.3% reduction. (b) Semi-logarithmic representation demonstrating exponential decay behavior of resistance with increasing temperature. (c) Arrhenius plot ($\ln(R)$ vs. $1000/T$) revealing linear behavior ($R^2 = 0.9982$) with activation energy $E_a = -0.026$ eV. The negative activation energy and a pre-exponential factor of 3.215×10^{-5} kΩ indicate a semiconductor-like thermal response. The Arrhenius plot's high linearity suggests a single conduction mechanism across the measured temperature range.

3 Characterization of the CS-Proteined Complex: Chemical and Physical Properties

The UV-Vis spectrum of the CS-proteinoid complex (Figure S4) shows significant absorption only in the violet region (380-400 nm). There are multiple peaks at 320.0, 321.1, 322.6, 324.2, 326.3, and 352.0 nm. These peaks indicate electronic transitions associated with the peptide bonds. Beyond the violet region, the spectrum has near-zero absorption. It transmits across the entire visible spectrum (400-750 nm) and extends into the near-infrared. The maximum trans-

mission of 1.66 occurs at 320.0 nm, followed by a sharp transition to complete transparency. This optical behaviour suggests that the CS-proteinoid complex interacts mainly with UV radiation. It remains transparent to visible light. This is a signature of protein-based materials with specific peptide bond configurations.

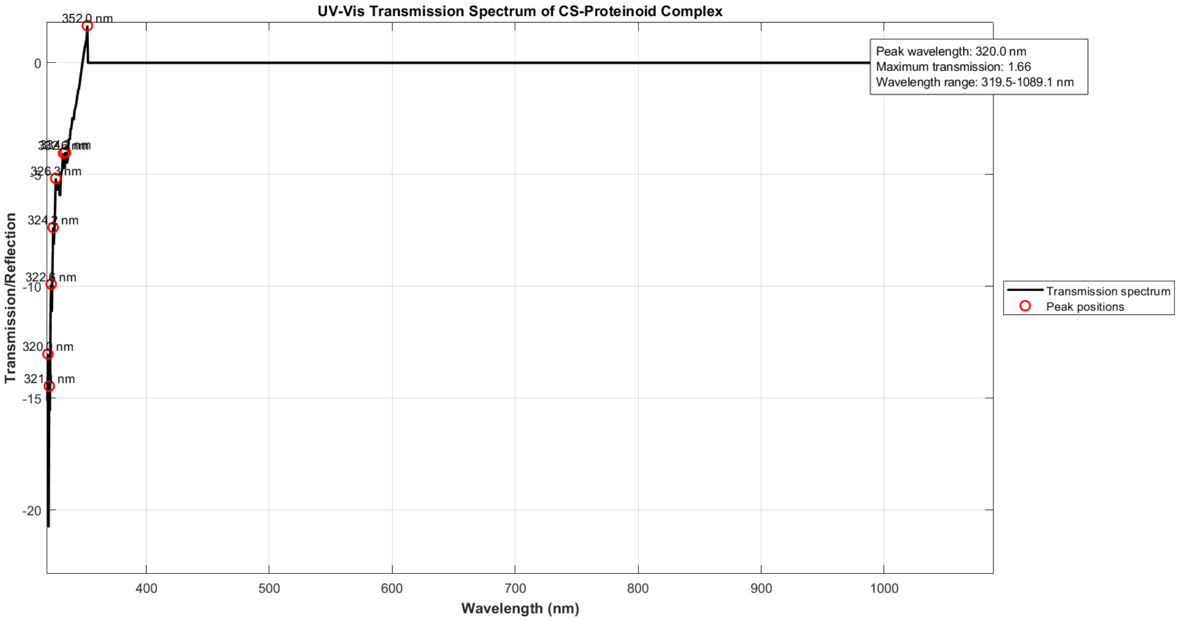

Figure S4: UV-Vis transmission spectrum of CS-proteinoid complex in the wavelength range of 319.5-1089.1 nm. The spectrum shows distinct absorption features in the UV region. It has peaks at 320.0, 321.1, 322.6, 324.2, 326.3, and 352.0 nm. These peaks indicate electronic transitions in the peptide bonds and aromatic amino acids. The sharp transition between 319-330 nm, followed by a plateau, shows the complex is optically transparent in the visible spectrum. A maximum transmission of 1.66 occurs at 320.0 nm. This suggests strong UV absorption, typical of protein-based complexes. The visible region (400-800 nm) has a stable baseline and clear peak positions. This shows a homogeneous CS-proteinoid complex with consistent optical properties.

Scanning electron microscopy showed the proteinoid microspheres' unique features, as in Figure S5. The SEM micrographs demonstrate successful formation of spherical structures with diameters ranging from ~0.5 to 2 μm. The microspheres exhibit a distinctive rough surface texture with interconnected structural features, indicative of the self-assembly process. The high-magnification images show the surface topology of individual microspheres. They reveal a uniform size and consistent shape across the sample. At $3,000\times$ magnification, the structure is clear. It confirms well-defined, spherical proteinoid complexes. This suggests that the CS-proteinoid material was successfully synthesized and assembled. The surface roughness and features in these microspheres are typical of proteinoid self-assembly. This indicates optimal reaction conditions during synthesis.

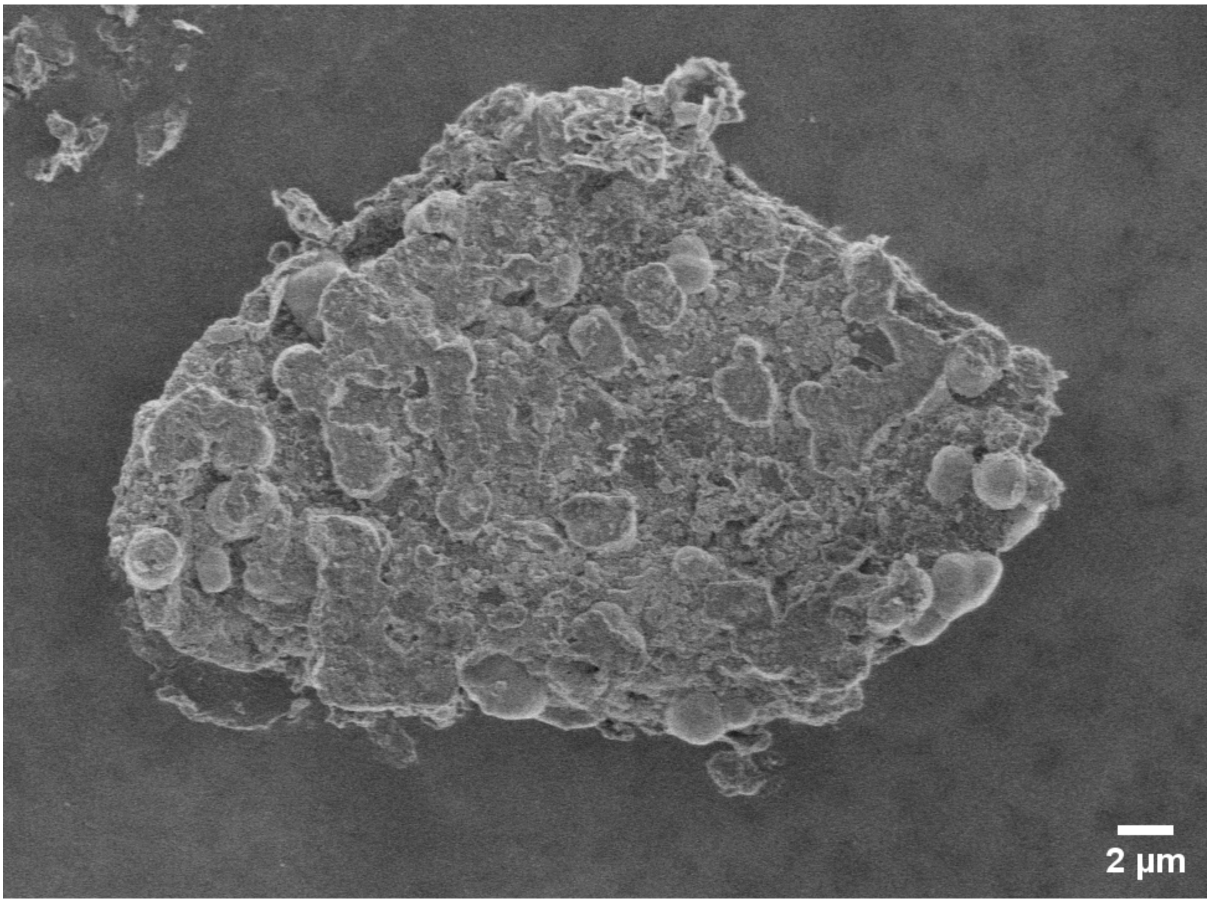

Figure S5: SEM images of proteinoid microspheres: An overview at $3,000\times$ magnification. They show the morphology and distribution of microspheres (scale bar: 10 μm), revealing detailed surface topology and spherical structures with diameters of $\sim$0.5-2 μm (scale bar: 2 μm). The images were acquired at a 1.60 kV accelerating voltage under high vacuum (1.36×10^{-5} Torr). The microspheres have a rough surface and interconnected features, indicating the successful formation of self-assembled proteinoid complexes. Operating conditions: WD = 2.6 mm, HFW = 69.1 μm, spot size = 1.6.
